# Inducing Effects of Illegal Drugs to Improve Mental Health by Self-Regulation Therapy: A Pilot Study

**DOI:** 10.3390/ijerph181910387

**Published:** 2021-10-02

**Authors:** Salvador Amigó

**Affiliations:** Department of Personality, Evaluation and Psychological Treatments, Faculty of Psychology, University of Valencia, 46010 Valencia, Spain; salvador.amigo@uv.es

**Keywords:** self-regulation therapy, coping strategies, emotionality, drugs

## Abstract

Background: This study consists of a brief psychological intervention, which uses Self-Regulation Therapy (SRT, procedure based on suggestion and classical conditioning), to improve coping with stress and emotionality by reproducing the positive effects of illegal drugs: cannabis, cocaine, ecstasy. Method: 15 volunteers (8 males, 7 females), with a mean age of 24.6 (SD = 4.4), underwent intervention to improve their coping with stress and emotionality using SRT. They carried out pre- and post-intervention scores for 10 days and during a 4-week follow-up. The employed instruments were: BSS (Barber Suggestibility Scale); COPE (Coping Skills Inventory), and PANAS (Positive and Negative Affect Schedule). Results: SRT was superior to non-intervention for the 4 coping strategies (*η*^2^ = 0.829, 0.453, 0.411 and 0.606) and for positive (*η*^2^ = 0.371) and negative emotionality (*η*^2^ = 0.419). An improvement in scores was evidenced in the follow-up scores compared to the pre-intervention measures. Conclusions: This study shows for the first time that it is possible to use illegal drugs, considered harmful to public health, to improve young people’s coping capacity and emotionality by reproducing their positive effects with SRT.

## 1. Introduction

A broad international consensus has been reached on the dangerousness and harmful effects of drugs for public health, especially illegal drugs [1,2]. Those drugs whose effects will be used in this study, cannabis, cocaine and ecstasy, are currently considered narcotics, and are subject to international control and classification. Cocaine and cannabis are classified in schedule I of the 1961 Single Convention on Narcotic Drugs, and are considered substances that are highly addictive and liable to abuse. Cannabis has also been classified in schedule IV until very recently, and is considered to have particularly dangerous properties, and little or no therapeutic value. Ecstasy is classified in schedule I of the Convention on Psychotropic Substances of 1971, and is considered a drug that presents a high risk of abuse, and poses a particularly serious threat to public health with little or no therapeutic value [3].

It is evident that illegal drugs are used because of their positive effects. For example, Boys et al. [4] found that the most popular functions for using drugs, such as cannabis, cocaine and ecstasy, were to relax (96.7%), become intoxicated (96.4%), keep awake at night while socializing (95.9%), enhance an activity (88.5%), and alleviate depressed moods (86.8%).

Would it be possible to find a way to take advantage of the positive effects of drugs by eliminating or reducing their negative effects at the same time? There is a psychological technique that has provided this possibility: Self-Regulation Therapy (SRT).

SRT is a psychological procedure created by Amigó in the 1990s [5], based on suggestion from the cognitive-behavioral perspective of hypnosis, and was especially designed to reproduce drug effects (for a review of the theoretical foundations and main applications of this procedure, see [6]).

Briefly, in the SRT, several sensory recall exercises are used to teach subjects how to voluntarily reproduce various physical sensations that are initially provoked by real stimuli. These sensations are associated with cues so that subjects are able to reproduce the effects later on, only with their imagination. At the end, participants are told that, as they have previously performed exercises, their minds are highly activated and receptive, which means that they can respond to the therapist’s verbal suggestions without having to be trained for each new session. During subsequent sessions, the entire procedure is shortened. The procedure is described in detail elsewhere [7].

The background of the SRT is very scarce as only few papers exist about the voluntary reproduction of the effects of drugs using suggestion; they are old publications and case studies with no experimental basis. In the very few cases about the clinical use of drug effects, it is only applied to treat addictions or as Psychedelic-Assisted Psychotherapy for emotional disorders (for a detailed review, see [6]).

SRT is the first psychological procedure based on reproducing the effects of drugs whose effectiveness during single sessions in reproducing such diverse drugs as heroin, cocaine, ecstasy, cannabis, amphetamine and methylphenidate has been well demonstrated [7,8,9]. Seeing as SRT can be used to improve positive moods and to reduce negative ones, it has been successfully used to treat patients with stress, anxiety and depression. However, there are only two published cases: one is a case study [10] and the other one is a single case experimental design [11].

This article shows, for the first time, the experimental application of a brief psychological intervention in a group of volunteers, using SRT to improve not only their ability to cope with stress, but also positive emotionality, while reducing negative emotionality. We used a within-subjects experimental design with a group of 15 young people who were taking drugs such as cannabis, cocaine and ecstasy. They had to reproduce these drug effects using SRT to improve their coping skills with stress and their emotionality.

It needs to be emphasized that this is a brief intervention whose real objective is to produce a rapid intense feeling of well-being, specifically to improve and strengthen coping capacity and emotionality during a short period of time, which may well be very useful in a specific situation of crisis, insecurity or low mood, and it is not a prolonged psychotherapy employed to solve or deal with all the participants’ problems or pathological symptoms. The participants or patients can repeat, and thus strengthen training as often as necessary. Obviously, it is a type of intervention that can be used very profitably to support conventional psychotherapy.

No patients participated; students and workers were the volunteers. This procedure can be considered part of the movement of positive psychology [12]. These authors focus on psychological interventions that increase individual happiness, and they found that several positive interventions lastingly increased happiness and decreased depressive symptoms. 

The brief psychological intervention herein used is based on two therapeutic programs: (1) Enhance positive emotions in your life and better cope with stress [7]; (2) Increase positive emotions with a suggestion and drugs program [13]. It is an adaptation that focuses, above all, on managing strategies for coping with stress and positive and negative emotions, which reveals an important field of clinical applications. This approach emphasizes the application of a personalized intervention (different drugs chosen by each participant, as well as the type of problems they want to face), with collecting intensive measures data prior and posterior to therapy in order to obtain its dynamic evolution [14].

The hypotheses of this study are: (1) participants will be able to improve coping skills with a more positive view of problems, a more active approach to them, and a better feeling of personal growth; (2) they will also be able to increase their positive emotionality and to reduce their negative one.

## 2. Materials and Methods

### 2.1. Participants

This study included 15 participants (8 males, 7 females), who were students (40%) and employees (60%) from the city of Valencia and cities in eastern Spain. Their mean age was 24.6 (SD = 4.4) years old and their age range was 20–34 years old.

Drug users were sought and, as we will see later on, those who responded sufficiently to the general suggestions and drug effects were subsequently selected.

### 2.2. Instruments

Substances Use Scale [15]. This instrument follows European Monitoring Centre for Drugs and Drug Addiction (EMCDDA) criteria and is a brief self-report questionnaire, which measures the frequency of drug use (such as cannabis, alcohol, tobacco, cocaine, MDMA, sedatives, hallucinogens and amphetamines).

Barber Suggestibility Scale (BSS) [16]. To evaluate the participants’ suggestibility level, the Spanish translation and adaptation prepared by [17] of the BSS was used. The BSS was designed to be administered individually, and is flexible in its use because it can be administered with or without hypnotic induction, and can be scored objectively or subjectively (OS and SS, respectively). This scale is composed of eight standardized test suggestions as follows: Arm Lowering, Arm Levitation, Hand Lock, Thirst “Hallucination,” Verbal Inhibition, Body Immobility, “Posthypnotic-Like” Response, Selective Amnesia. In a Spanish sample composed mainly of students, α = 0.76 was obtained for OS [18]. Reliability indices were also obtained in the clinical population [19]: OS (α = 0.70); SS (α = 0.80).

COPE Inventory [20]. COPE is a 60-item Likert-type inventory. The scale score goes from 1 (no effect) to 4 (maximum effect). This is a multidimensional coping inventory to assess the different ways in which people respond to stress. This instrument was designed to assess 15 conceptually distinct coping methods. We used the situational format. The instructions for this version ask the respondents to indicate the extent to which they have been engaged in each coping response during a particular period of time. We obtained COPE ratings using two different temporal instructions. The subjects rated how they felt: (a) “today” (Today) and (b) “during the past week” (Past Week). We used the Spanish adaptation of [21]. COPE is composed of 15 scales. For the purposes of this study, four scales were chosen: a) Planning and Active Coping (6 items), α = 0.78; b) Positive Reinterpretation (3 items), α = 0.64; c) Personal Growth (2 items), α = 0.60; d) Behavioral Disengagement (3 items), α = 0.75.

Positive and Negative Affect Schedule (PANAS) [22]. This scale consists of a number of words that describe different feelings and emotions, and comprises two 10-item mood scales on the Positive and Negative Affect. As with COPE, we obtained PANAS ratings using two different temporal instructions. The subjects rated how they felt: (a) “today” (Today) and (b) “during the past week” (Past Week). We used the Spanish adaptation from Sandin et al. [23]. Cronbach’s coefficients were high, for both men (α = 0.89 (PA), α = 0.91 (NA)), and women (α = 0.87 (PA), α = 0.89 (NA)). 

### 2.3. Procedure

All sessions, including the first informative meeting, took place in rooms and offices of the Faculty of Psychology of the Valencia University. A written announcement was published, and information was offered in some classrooms about the study to recruit volunteers. Contact was maintained mainly by e-mail.

The requirements to be admitted in the study were: being an occasional user of an illegal drug (in the evaluation phase) and having a sufficient level of suggestibility and ability to reproduce drugs with suggestion (in the training phase). We will come back to this later on.

Regarding the exclusion criteria, these were: being a frequent user of illegal drugs and receiving psychological treatment.

On the other hand, the admitted participants were asked to look for new volunteers in the cities where they resided, both students and workers, which constitutes a “snowball” method to obtain part of the sample.

Then, first, an informative meeting was held in order to collect epidemiological and drug use information and was when informed consents were signed. In addition, the participants filled in the COPE and PANAS scales. The subjects rated how they coped with stress and felt during the past week. This was the pre-intervention record (Pre-COPE and Pre-PANAS). The participants filled out the forms in paper in every phase of this study.

Afterward, they were asked to fill in the COPE and PANAS scales at home for 10 days in relation to how they felt at the end of the day. The records of these 10 days were considered the Control Condition (CC).

After completing the 10 days, three training sessions with SRT were held. In the first training session, the suggestibility level and ability to reproduce drug effects were evaluated. To measure the level of suggestibility, objective and subjective BSS scores were obtained. At this time, new exclusion criteria (see above) were used: not reaching medium-low levels of suggestibility (BSS), not reproducing at least three sensations of the drug chosen during the first reproduction of drug effects. In this case, 3 participants out of 18 were excluded.

The objective of the three training sessions with SRT was to improve the reproduction of the chosen drug (11 chose cannabis, 2 cocaine and 2 ecstasy), the ability to use these effects therapeutically (improve coping with problems and emotionality) and the capacity to apply the technique autonomously at home and in any other circumstance or place. All the three SRT training sessions also took place in the same rooms on three consecutive days throughout the same week.

Afterward, they should practice it at home for 10 days (Intervention Condition, IC) by filling in the COPE and PANAS scales at the end of the day and reflecting on how they have behaved and felt all day. On the fourth and eighth days during the 10-day intervention phase in which the participants practiced SRT at home, two supervision sessions were held. These sessions took place in the Faculty of Psychology rooms, and the researcher checked how they worked alone at home and how they practiced doing that in every place and circumstance in their everyday lives.

Finally, after they completed the intervention condition for 10 days, a month follow-up was carried out. The subjects had to fill in the COPE and PANAS scales at home for 4 weeks (at the end of each of the four weeks) by answering how they felt and behaved throughout each week. It took the same response format as the pre-intervention record. A schematic of the procedure is presented in Table 1 for clarity.

### 2.4. Experimental Design

This study uses a single-group interrupted time-series design with 10 scores per condition (control and intervention). Sampling was not random due to the nature of this study. Therefore, this is a quasi-experimental design. The commonest threats to internal validity in this kind of design are: history, selection, instrumentation, regression, maturation [24]. Certain conditions can reduce these threats: treatment onset is immediate, temporal time intervals are short, treatment effect is immediate, and the effect is large in relation to prior intertemporal variation [25]. Several controls were established to improve the experimental design and considerable effort has been made in this study to reduce all of these threats, therefore, the demanding conditions above cited were what our experimental design meets.

### 2.5. Statistical Analysis

Data were analyzed using IBM Corp., released 2015, IBM SPSS Statistics for Windows, Version 26.0., IBM Corp (Armonk, NY, USA). General Linear Model and non-parametric statistics from SPSS were also used (Friedman and Wilcoxon Signed Rank Test). 

Two-way Repeated Measures ANOVA from the General Linear Model statistical procedure were performed. The two factors were: Time (10 levels) and Intervention (2 levels, CC and IC).

To carry out and interpret the analyses, the used criteria were the following: (1) If the hypothesis of sphericity was not rejected, we chose the univariate F statistic of assumed sphericity, as in this case, it is the most powerful test, especially for small sample sizes; (2) If the hypothesis of sphericity was rejected, we chose the F value by applying a correction index called epsilon, either the Greenhouse–Geisser or Huynh–Feldt estimation, depending on the highest value of power. If power was the same, the most conservative estimate was chosen, which was Greenhouse–Geisser; (3) In the event of extreme non-compliance with the assumption of sphericity, the Lower Limit estimator was chosen; (4) We omitted presenting the identification of all these tests in the tables for space reasons; (5) The degrees of freedom for intervention were 1, and for time and interaction, were 9.

The choice of the repeated measures ANOVA statistic is appropriate in this study due to the nature of the design and the data, and although a larger sample size is desirable, this test is not particularly disadvantaged with a small sample if the data number is the same in the control and intervention condition [26]. On the other hand, and as it will be seen later, the power observed is high for the intervention effect.

## 3. Results

The drugs most frequently used by the participants in the present study last year were: alcohol (*n* = 15), cannabis (*n* = 15), tobacco (*n* = 12) and tranquilizers (*n* = 11). In the last month they were: cannabis (*n* = 14), alcohol (*n* = 14) and tobacco (*n* = 10).

The participants chose the drug they wished to reproduce with the SRT: eleven chose cannabis, two cocaine and two ecstasy.

Table 2 shows the ANOVA results for the COPE and PANAS variables. We can observe that SRT had a statistically significant effect for the four coping strategies, but time also had a significant effect for Planning and Active Coping and Personal Growth.

Hence intervention had a significant main effect (F(1,14) = 68.01; *p* < 0.001; MSE = 20.26; *η*^2^ = 0.829) on Planning and Active Coping, as did time (F(9, 111.35) = 3.24; *p* < 0.05; MSE = 3.71; *η*^2^ = 0.188). The effect of the interaction was not significant.

Intervention also had a significant main effect (F(1,14) = 11.59; *p* < 0.05; MSE = 9.21; *η*^2^ = 0.453) on Positive Reinterpretation, while the effects of time and interaction were not significant.

Intervention had a significant main effect on Personal Growth (F(1,14) = 9.75; *p* < 0.05; MSE = 5.25; *η*^2^ = 0.411), as did time (F(9, 126) = 1.98; *p* < 0.05; MSE = 0.64; *η*^2^ = 0.124), while the effect of interaction was not significant.

For Behavioral Disengagement, a significant main effect was observed with intervention (F(1,14) = 21.49; *p* < 0.001; MSE = 17.71; *η*^2^ = 0.606), whereas the effects of time and interaction were not significant.

With the PANAS scales, intervention had a significant main effect (F(1,14) = 8.26; *p* < 0.05; MSE = 119.86; *η*^2^ = 0.371) on the PA schedule, as did time (F(8.208, 114.91) = 2.648; *p* < 0.05; MSE = 30.58; *η*^2^ = 0.161), but the effect of the interaction was not significant. Moreover, intervention had a significant main effect (F(1,14) = 10.08; *p* < 0.05; MSE = 110.77; *η*^2^ = 0.419) on the NA schedule, with insignificant effects for time and interaction.

Table 3 shows only the results of the ANCOVA which were significant when the pre-intervention variables of both COPE and PANAS were included. The effect of time on Planning and Active Coping strategy became non-significant, while the interaction of the pre-intervention covariate with the strategy itself was significant. A significant effect of SRT was observed for the Positive Reinterpretation and a significant interaction with the pre-intervention score in the same strategy (*p* < 0.05), that is, the favorable predisposition to use positive restructuring strategies the week before the study started influenced the therapeutic success.

**Figure 1:**
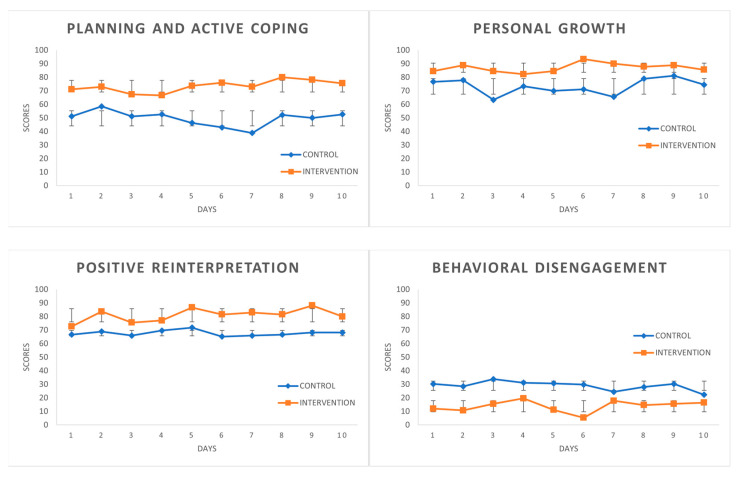
COPE Variables in Control Condition and Intervention Condition. Mean values (SD).

**Figure 2:**
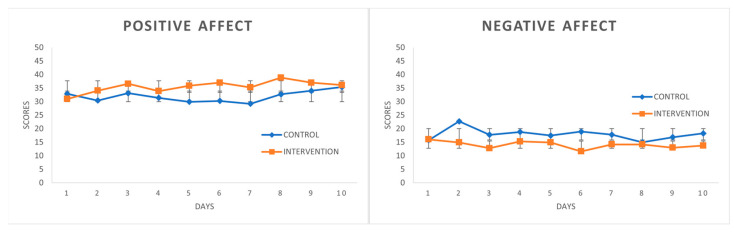
PANAS variables in control condition and intervention condition. Mean values (SD).

Figure 1 depicts how the scores during the CC (control condition) represent a very stable curve and how the scores during the IC (intervention condition) are clearly higher for the first three coping strategies and are lower for Behavioral Disengagement. Stable curves were observed for the PANAS scales in CC (see Figure 2). Moreover, the PA scores slightly rose on the last days. Even so, the two scales followed the expected curve in IC, with PA above the CC curve in most scores displaying an upward trend, while the NA scores in IC were below no treatment.

**Figure 3:**
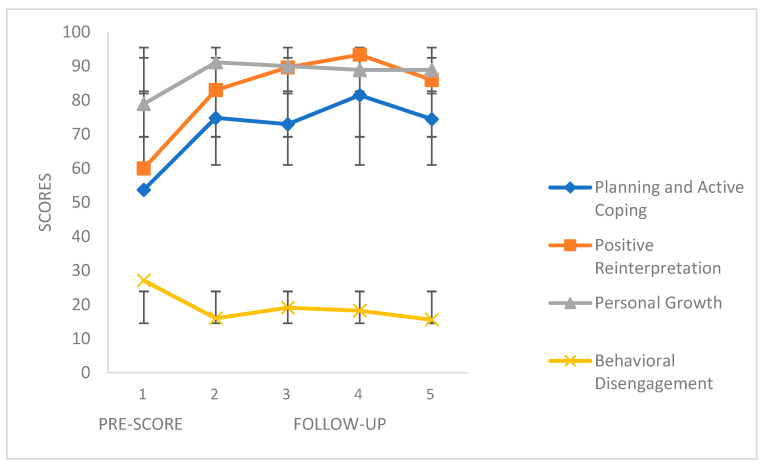
COPE variables in the follow-up. Mean values (SD).

**Figure 4:**
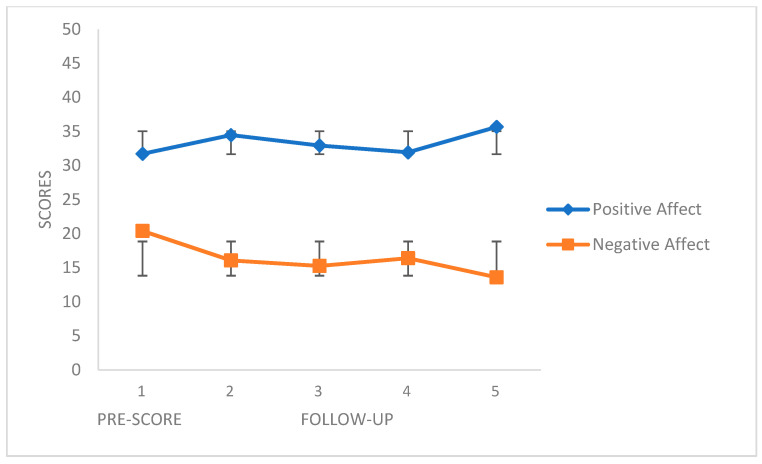
PANAS variables in the follow-up. Mean values (SD).

Figure 3 and Figure 4 show the follow-up scores of the four coping strategies and the PANAS scales, respectively. The COPE scales revealed different score ranges (distinct numbers of items), and to match them, ipsative scores were obtained to better clarify the interpretation of the figures. The first point represents the score before the beginning of the first phase (CC), and the remaining four points represent the scores of the 4-week follow-up. We can see how the positive coping skills (Planning and Active Coping, Positive Reinterpretation and Personal Growth) increased during the follow-up, while the negative coping strategies (Behavioral Disengagement) obtained lower scores. The same applies the PANAS Positive Affect and Negative Affect scales, respectively.

Some statistical analyses were carried out about follow-up data. The results are shown in Table 4.

Friedman test, as the non-parametric alternative to the one-way ANOVA with repeated measures, was used for pre-score (time 0) and for follow-up (4 points, each one for a week). There was a statistically significant difference in Planning and Active Coping (Χ^2^ = 19.78; *p* < 0.01), Positive Reinterpretation (Χ^2^ = 25.32; *p* < 0.001), Positive Affect (Χ^2^ = 11.76; *p* < 0.05), and Negative Affect (Χ^2^ = 14.06; *p* < 0.01).

Post hoc analysis with Wilcoxon signed-rank tests was conducted to the variables with significant Χ^2^, with a Bonferroni correction applied (0.05/4), resulting in a significance level set at *p* < 0.012. Comparisons between point 0 (pre-score) and the 4 follow-up points were carried out.

There was a statistically significant increase in Planning and Active Coping and Positive Reinterpretation in all of the 4 follow-up points (*p* < 0.012 in every point), and a significant reduction in the last follow-up point in Negative Affect (*p* < 0.012).

In general, we found significant and satisfactory results in the follow-up, but only for the Planning and Active Coping, Positive Reinterpretation and Negative Affect variables.

## 4. Discussion

This is the first study to show the efficacy of a brief intervention based on reproducing the effects of illegal drugs, such as cannabis, cocaine and ecstasy, to improve coping skills and emotionality, and to increase positive emotionality and reduce negative emotionality. Thus the hypotheses put forward at the beginning are confirmed.

In recent times, research that explored the impact of psychedelics has emerged, such as lysergic acid diethylamide (LSD) and psilocybin in psychotherapy (for reviews, see [27,28,29,30,31]).

However, recent research on psychedelics and hypnosis has appeared largely in isolation. The potential of harnessing the power of suggestion to influence the response to psychedelics may have implications for both clinical and basic research [32]. These authors found commonalities and differences between psychedelics and hypnosis that indicate the potential efficacy of combining both in psychotherapy, and they suggest a plan guide and integration of the psychedelic experience in order to enhance therapeutic outcomes.

Yet, all of these studies are based on the Psychedelic-Assisted Psychotherapy approach. As Lemercier and Terhune [32] stated, one potential benefit of combining psychedelics and hypnosis could be to use suggestions to reproduce such experiences on the days following administration of psychedelics. Nonetheless, very few studies about this can be found, and they all indicate a single session (see [33,34]).

SRT is a psychological procedure based on suggestion without hypnosis with proven efficacy in reproducing many different drug effects. It has also been used as a therapeutic technique to treat psychopathological symptoms in patients, such as anxiety or depression, by reproducing ephedrine [11], and also improving coping skills and positive emotionality with methylphenidate [10]. However, these two studies were conducted with a single patient each according to a single case experimental design and a case study, respectively, and they were non-drug users who reproduced the effects they experienced with legal drugs. This article attempts to move one step further by checking the effectiveness of SRT by increasing coping skills and emotionality by a detailed protocol and intra-group design. This was to increase the more positive view of problems, the ability to plan and to actively cope with problems, and to reduce the tendency to avoid them, as well as the experience of personal growth. The participants increased positive emotionality and reduced negative emotionality. These improvements remained, and increased in some cases, over a 4-week follow-up. SRT can also take advantage of the drug’s power itself so that drugs can also increase hypnotic susceptibility. So, methylphenidate enhancement of hypnotizability in adults with ADHD [35] and a low dose of ketamine in healthy volunteers can increase not only the subjective ratings of dissociation but also hypnotizability [36]. This has already been proven with SRT [9].

This study has clear limitations that must be corrected in future studies. Thus, including more participants is required as is the inclusion of a control group and a longer follow-up period. However, a large effort has been made to reduce internal validity, just as we stated in Section 2.4. On the other hand, as previously stated, the real objective of this intervention was to improve and strengthen coping capacity and emotionality during a short period of time. Another limitation may be ethical in nature. If the participants reproduce the effects of illegal drugs, could this not lead them to use those drugs in greater quantities? In this study, participants were asked to report any negative effects that the procedure might have produced on them in the months following its completion. No one reported adverse effects. On the contrary, there is clinical evidence and research results that indicate that this procedure can reduce craving and the urge to use the drugs. This was proven with cocaine and heroin users, where they were able to reduce both craving and drug use. Participants said that if they were able to produce the effects of drugs “mentally” they did not need to consume them. Therefore, SRT may also represent a therapeutic potential for the treatment of addictions [37,38]. However, more research is needed about this. We also added an analysis of covariance (ANCOVA), which strengthened our design by enhancing the possibility of rejecting the null hypothesis [39]. Thus, we observe that willingness to use certain coping strategies prior to intervention influenced the intervention results. More modulating variables should be considered in the future. Regarding the possible bias in the selection of the participants, it must be recognized that the impossibility of using these drugs for legal reasons entails having to form this type of sample, with a certain level of suggestibility and the ability to reproduce drug effects. As discussed above, the SRT is also useful for reproducing effects of those drugs administered for this purpose.

Therefore, the results of this study do not provide definitive evidence about its efficacy; of course, because it is a pilot study with a small sample, and although a big effort has been made to reduce the threats to internal validity of the present design (as it has been above explained), a designed crossover randomized controlled trial is needed. However, this study can be considered as a first step in a novel type of psychological brief intervention using an innovative approach in such a way that uses illegal drugs that it could not be used in any other ways.

## 5. Conclusions

The fact that the effects produced by illegal drugs, such as those herein considered (cannabis, cocaine, ecstasy) can have clinical applications if psychological procedures like SRT are employed, makes it reasonable to assume a strong impact in the psychotherapy and drug policy context.

Some alternative proposals exist for classifying drugs [40]. In this new classification, alcohol comes over as the most harmful drug, followed by heroin and crack cocaine. The other drugs (e.g., cocaine, cannabis, ecstasy) are shown as being less harmful. If we also consider that it is possible to take advantage of their effects with psychological techniques such as SRT, then the reason for classifying them as dangerous and with little or no therapeutic value does not hold.

In short, and as far as we know, this is the first experimental confirmation following an intra-group design of the efficacy of combining the effects of illegal drugs with suggestions to improve human potentialities, such as coping skills and emotionality, which opens up an unusual field of clinical applications and can have a clear impact on new international drug policies.

## Figures and Tables

**Table 1:** Procedure scheme.

Evaluation	Control Condition (CC)	Training Sessions	Intervention Condition (IC)	Follow-Up
Epidemiological data Pre-Questionnaires: ▪ Substances Use Scale ▪ PANAS ▪ COPE Informed consent signatures. Apply inclusion and exclusion criteria for this phase.	Objective: Fill in two questionnaires at the end of the day over a period of 10 days. Instruments: ▪ PANAS ▪ COPE	Objective: Assess hypnotic susceptibility with BSS (OS and SS). Learn Self-Regulation Therapy (SRT) and be able to reproduce the effects of drugs. Apply the exclusion criteria for this phase.	Objective: Apply at home for 10 days the reproduction of the positive effects of drugs with SRT, and fill in two questionnaires at the end of the day. Instruments: ▪ PANAS ▪ COPE It includes 2 supervision sessions.	Objective: Keep the effects for 1 month. Fill in two questionnaires at the end of each week. Instruments: ▪ PANAS ▪ COPE

BSS: Barber Suggestibility Scale; OS: Objective Score; Subjective Score; SRT: Self-Regulation Therapy; COPE: Coping Inventory; PANAS = Positive and Negative Affect Schedule.

**Table 2:** Two-way repeated measures ANOVA of COPE and PANAS variables.

	Factors	Sig. Mauchy’s W	F	Df	Mean Square Error	Df of MSE	Sig.	Partial Eta Squared	Observed Power
PAC	Intervention	–	68.01	1	20.26	14	0.000	0.829	1.000
	Time	0.01	3.24	9	3.71	111.35	0.002	0.188	0.962
	Interaction	0.00	3.03	1	39.31	14	0.103	0.178	0.368
PR	Intervention	–	11.59	1	9.21	14	0.004	0.453	0.886
	Time	0.08	1.47	9	1.46	126	1.63	0.095	0.681
	Interaction	0.24	0.730	9	1.65	126	0.680	0.50	0.348
PG	Intervention	–	9.75	1	5.25	14	0.007	0.411	0.827
	Time	0.13	1.98	9	0.64	126	0.047	0.124	0.831
	Interaction	0.00	1.43	1	0.77	14	0.250	0.093	0.201
BD	Intervention	–	21.49	1	17.71	14	0.000	0.606	0.990
	Time	0.00	1.18	1	29.21	14	0.295	0.078	0.174
	Interaction	0.05	1.74	9	3.30	126	0.085	0.111	0.769
PA	Intervention	–	8.26	1	119.86	14	0.012	0.371	0.762
	Time	0.03	2.64	8.20	30.58	114.91	0.009	0.161	0.922
	Interaction	0.00	1.65	1.00	50.97	84.19	0.220	0.106	0.224
NA	Intervention	–	10.08	1	110.77	14	0.007	0.419	0.839
	Time	0.00	2.07	1.00	192.77	14.00	0.172	0.129	0.269
	Interaction	0.00	2.12	1.00	205.39	14.00	0.167	0.132	0.274

COPE = Coping Skills inventory; PANAS = Positive and Negative Affect Schedule; PAC = Planning and Active Coping; PR = Positive Reinterpretation; PG = Personal Growth; BD = Behavioral Disengagement; PA = Positive Affect; NA = Negative Affect; *Df* = Degrees of freedom. The observed power has been calculated using alfa = 0.05.

**Table 3:** Two-way repeated measures ANCOVA of some coping strategies with pre-intervention.

	Factors	Sig. Mauchy’s W	F	Df	Mean Square Error	Df of MSE	Sig.	Partial Eta Squared	Observed Power
PAC	Pre-PAC		5.68				0.033	0.304	0.998
	Intervention	–	15.27	1	243.11	13	0.002	0.540	0.950
	Intervention * Pre-PAC		4.81	1	76.71		0.047	0.270	0.529
	Time	0.05	0.55	9	1.85	117	0.828	0.041	0.263
	Interaction	0.03	1.33	5.99	39.31	77.93	0.250	0.093	0.494
RP	Pre-RP		9.61				0.008	0.425	0.817
	Intervention	–	9.79	1	70.09	13	0.008	0.430	0.824
	Intervention * Pre-RP		5.01	1	35.91		0.043	0.279	0.545
	Time	0.12	1.44	9	2.18	117	0.178	0.100	0.666
	Interaction	0.10	0.70	9	1.17	117	0.701	0.052	0.335

PAC = Planning and Active Coping; PR = Positive Reinterpretation; *Df* = Degrees of freedom. The observed power has been calculated using alfa = 0.05.

**Table 4:** Statistical tests (Friedman and Wilcoxon) for follow-up points and first point pre-intervention for COPE and PANAS variables.

Variables	Pre- and Follow-Up Scores (Friedman)		Pear Comparison (Wilcoxon)							
	Χ^2^	*p*	0–1		0–2		0–3		0–4	
			Z	*p*	Z	*p*	Z	*p*	Z	*p*
Planning and Active Coping	19.78	0.001	−2.62	0.009	−2.79	0.005	−3.24	0.001	−2.93	0.001
Positive Reinterpretation	25.32	0.000	−2.63	0.008	−2.99	0.003	−3.21	0.001	−2.79	0.007
Personal Growth	4.60	0.331	-	-	-	-	-	-	-	-
Behavioral Disengagement	9.46	0.51	-	-	-	-	-	-	-	-
Positive Affect	11.76	0.019	−1.10	2.71	−0.50	0.61	−0.21	0.834	−2.04	0.041
Negative Affect	14.06	0.007	−1.42	0.15	−1.75	0.070	−1.82	0.084	−2.81	0.005

0: Pre-intervention point; Follow-up points: 1 (first week), 2 (second week), 3 (third week), 4 (fourth week).

## Data Availability

The data presented in this study are available on request from the corresponding author. The data are not publicly available due to ethical consideration and to preserve the privacy of the participants.
